# Myco-Ed: Mycological curriculum for education and discovery

**DOI:** 10.1371/journal.ppat.1013303

**Published:** 2025-07-18

**Authors:** Sara Branco, Peter G. Avis, Kerrie Barry, Scott Bates, Gerald M. Cobián, Ellen G. Dow, Sara Gremillion, Amy Honan, Chinyere A. Knight, Kurt LaButti, C. Alisha Quandt, Jane E. Stewart, Jayson Talag, Andrew W. Wilson, Lotus Lofgren, Stephen James Mondo

**Affiliations:** 1 Department of Integrative Biology, University of Colorado Denver, Denver, Colorado, United States of America; 2 School of Biology and Ecology, University of Maine, Orono, Maine, United States of America; 3 DOE Joint Genome Institute, Lawrence Berkeley National Laboratory, Berkeley, California, United States of America; 4 Department of Biological Sciences, Purdue University Northwest, Hammond, Indiana, United States of America; 5 Department of Biological Sciences, California State University Chico, Chico, California, United States of America; 6 Environmental Genomics and Systems Biology Division, Lawrence Berkeley National Laboratory, Berkeley, California, United States of America; 7 Department of Biology, Georgia Southern University, Savannah, GeorgiaUnited States of America; 8 Department of Botany and Plant Pathology, Oregon State University, Corvallis, Oregon, United States of America; 9 Department of Biology, Tuskegee University, Tuskegee, Alabama, United States of America; 10 Department of Ecology and Evolutionary Biology, University of Colorado Boulder, Boulder, Colorado, United States of America; 11 Department of Agricultural Biology, Colorado State University, Fort Collins, Colorado, United States of America; 12 Arizona Genomics Institute, University of Arizona, Tucson, Arizona, United States of America; 13 Department of Research and Conservation, Denver Botanic Gardens, Denver, Colorado, United States of America; 14 Department of Plant and Microbial Biology, University of California Berkeley, Berkeley, California, United States of America; University of Michigan Health System, UNITED STATES OF AMERICA

## Abstract

Fungi are important and hyperdiverse organisms, yet chronically understudied. Most fungal clades have no reference genomes, impeding our understanding of their ecosystem functions and use as solutions in health and biotechnology. Also, opportunities for training in fungal biology and genomics are lacking, creating a bottleneck that hinders the recruitment and cultivation of a talented future mycological workforce. To address these issues, we developed Myco-Ed, an educational program offering training and scientific contributions through genome sequencing and analysis. Myco-Ed empowers students to pursue careers in fungal biology while improving fungal resources. Myco-Ed has been piloted at 12 institutions (15 classrooms) ranging from online e-Campuses to R1 universities, resulting in hundreds of fungal observations and many new high-quality reference genomes.

## Introduction

Fungi are hyperdiverse and understudied but play crucial roles in ecosystems [1–4]. They represent a huge swath of the world’s biological diversity, and yet, of the estimated 2–3 million fungal species, only a small fraction are currently known to science [2]. Fungi also produce valuable natural products, with many potentially useful metabolites remaining undiscovered [5], that can be crucial for addressing ongoing challenges of climate change, pollution, agriculture, ecosystem health, and medicine optimization [5–8]. Given the labor-intensive challenges of isolating understudied groups of fungi, there are still considerable gaps in genomic knowledge for many fungal taxa. The lack of high-quality fungal reference genomes hampers our ability to fully understand fungal roles in nature and realize their potential for applications in health, energy, industry, and ecosystem management. Furthermore, there is a strong need for basic training in microbiology [9] and a skilled workforce able to face the growing size and complexity of genomic data. Despite this overwhelming need, there are alarmingly limited opportunities for training in fungal biology and genomics, making talent recruitment challenging and delaying advancements in these disciplines [10].

**Fig 1:**
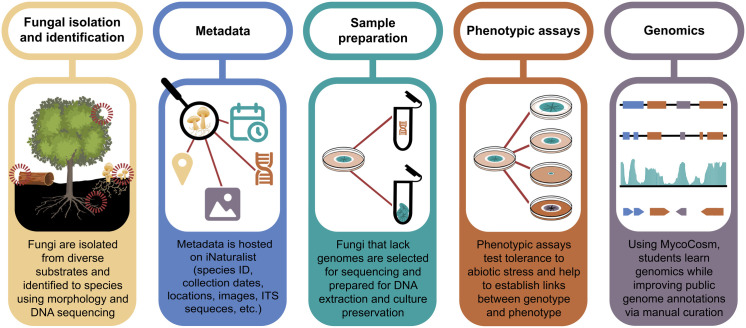
The Myco-Ed program is divided into modules that cover fungal isolation and identification, metadata collection, sample preparation, phenotypic assays, and curating fungal genome annotations.

To train a future generation of researchers and improve fungal genomic resources, we developed **Myco-Ed: The Mycological Curriculum for Education and Discovery** (https://mycocosm.jgi.doe.gov/mycocosm/home/myco-ed), a publicly available workforce development program designed for higher education courses (Fig 1). These modular hands-on experiments train students in laboratory techniques, bioinformatics, and data analysis. Throughout Myco-Ed, students isolate and identify fungi from the environment and prepare materials for production of novel fungal genomes. Students track their observations and compile metadata using iNaturalist [11], perform phenotypic assays and manually curate fungal genes. These data can then be tied to the newly generated Myco-Ed reference genomes, enabling genotype-phenotype prediction and improving the quality of publicly available fungal gene annotations. Myco-Ed partners with the Arizona Genomics Institute (AGI) and the Joint Genome Institute (JGI) to provide free-of-cost, high-quality DNA extractions, genome sequencing, assembly, and annotation. Reference genomes are shared in MycoCosm (https://mycocosm.jgi.doe.gov/mycocosm/home), a public web portal created and maintained by the JGI that provides data access, visualization, and analytical tools for comparative genomics of fungi [12]. In addition, cultures of the sequenced isolates will be deposited in culture collections, providing isolate reservoirs and an important community resource. Myco-Ed is currently funded through an ongoing JGI Director’s Science award, and we are actively seeking additional funding for long-term program support.

## Myco-Ed modules

Myco-Ed consists of publicly accessible, tested research modules and protocols, compiled and available through protocols.io (dx.doi.org/10.17504/protocols.io.rm7vzqd5xvx1/v1). These freely available resources can be implemented in any classroom or laboratory course, and instructors may implement the whole program or focus on select modules to fit their training or learning goals and course objectives.

*Fungal environmental sampling and identification.* Students isolate fungi from their local environment, compile metadata, and identify samples using the internal transcribed spacer (ITS) region of the nuclear RNA gene or other appropriate genetic markers. 

*Metadata repository*. Students record data on fungal isolates using iNaturalist. This free online social network records user metadata, including collection dates, locations, species identifications, images, DNA sequence data, phenotypic information, etc. An umbrella iNaturalist project houses all Myco-Ed data (https://www.inaturalist.org/projects/myco-ed-fungal-genomics-education-project). Individual classroom projects document all the information regarding the fungi collected, allowing for easy public data access and compilation.

*Selection*, s*ample preparation, and genome sequencing*. As part of their coursework, students amplify and sequence the fungal ITS region from cultured fungal isolates. A BLAST analysis against NCBI’s fungal ITS database is then performed to identify potential matches. The ITS sequences and best hits alignments are then submitted to Myco-Ed and screened at both NCBI and JGI to assess whether the student sample or a closely related species has previously been sequenced or is currently in progress. This evaluation ensures that we prioritize novel fungal genomes that contribute new insights into the understanding of fungal diversity and ecology, ultimately enhancing the scope of our research efforts. Tissue from selected fungi is then sent to the AGI which performs high-quality DNA extraction and sends extracts to the JGI. The genome is then sequenced at the JGI using the PacBio sequencing platform, assembled, and annotated. Students also prepare live cultures for future deposition at culture collections, such as the Fungal Genetics Stock Center, allowing for future studies on environmental isolates with available reference genomes.

*Fungal phenotypic assays*. Students also collect fungal phenotypic data that helps establish links between genotypes and phenotypes. For example, abiotic stress assays for metal and salt tolerance were performed, and the resulting data was deposited in iNaturalist.

*Genomics*. This module includes introductory comparative genomic exercises using MycoCosm. Students learn genomic analyses while improving genome annotations via manual curation, resulting in a more informative, reliable, and useful database for students and researchers. To date, genomic lab exercises included the determination of fungal mating types and the analysis of natural product distribution. Student curations are shared with the community through MycoCosm (available via search or through individual protein pages).

## Myco-Ed achievements

Within its first two years, Myco-Ed was implemented in 15 individual classrooms across the US (S1 Table), training 316 students in fungal biology, bioinformatics, and genomics, directly contributing to expanding needed mycological expertise. Myco-Ed students have had the opportunity to engage in ‘real-world’ scientific experiments, enabling them to participate in the scientific process and gain experience with experimental design. They have also developed skills in microbiological and molecular techniques as well as bioinformatics. Preliminary student survey data revealed positive program impacts, including an increase in student confidence in conducting research and an enhanced sense of identity and belonging within the scientific community. Most notable was an increase in students’ familiarity and ability to use bioinformatics to answer scientific questions after participation in the Myco-Ed curriculum. A larger-scale assessment of the program is planned for the future.

Furthermore, the program has already generated 16 new reference genomes (https://mycocosm.jgi.doe.gov/MycoEd/) and has 30 more in the works, including first references for several fungal species, genera, and possibly higher taxonomic levels (Table 1). Notably, we obtained chromosome-level assemblies for multiple samples, demonstrating that Myco-Ed is an effective way to generate high-quality reference genomes.

**Table 1:** Myco-Ed fungal reference genomes compiled to date, including organism name, strain ID, institution, project status, and novelty (the first genome at that taxonomic level). Novelty was determined by screening student isolates against NCBI and MycoCosm for available genomes. If the best hit for a student isolate was to a genus with a reference genome, but percent identity was below 95%, it was marked as Genus+ (potentially representing a new genus or higher taxonomic level). Projects with status ‘Complete’ are publicly available via MycoCosm (https://mycocosm.jgi.doe.gov/MycoEd), while those ‘In preparation’ have been selected for sequencing but have not yet been completed.

Organism name	Strain	Institution	Status	Novelty
*Alternaria* sp.	Myco 013-3.2	University of Colorado Denver	Complete	Species
*Aspergillus fumigatus*	P4 SB	University of Colorado Denver	Complete	Strain
Didymosphaeriaceae sp.	SO21a	Oregon State University	Complete	Genus+
*Epicoccum nigrum*	MEM15	Oregon State University	Complete	Strain
*Penicillium fagi*	P1 LN2	University of Colorado Denver	Complete	Species
*Penicillium ribium*	P3 LN2	University of Colorado Denver	Complete	Species
*Penicillium* sp.	P5 SB	University of Colorado Denver	Complete	Species
*Periconia celtidis*	DY2	Oregon State University	Complete	Species
Pleosporales sp.	UCDMyco 002 3.1	University of Colorado Denver	Complete	Genus+
*Preussia procaviae*	UCDMyco 009	University of Colorado Denver	Complete	Genus+
Sporormiaceae sp.	Myco 019 3.1	University of Colorado Denver	Complete	Genus+
*Trichoderma paraviridescens*	ED13	Oregon State University	Complete	Species
*Aleurodiscus thailandicus*	TULF-12	Tuskegee University	In preparation	Genus+
*Apiospora* sp.	DY4	University of Colorado Denver	In preparation	Genus+
Apiosporaceae sp.	Myco 0061	University of Colorado Denver	In preparation	Genus+
Apiosporaceae sp.	UCDMyco 010 3.1	University of Colorado Denver	In preparation	Genus+
*Briansuttonomyces eucalypti*	BS365-24-1	Colorado State University	In preparation	Genus+
*Diatrype lijangensis*	TULF-10	Tuskegee University	In preparation	Species
*Efibula americana*	TULF-14	Tuskegee University	In preparation	Genus+
*Heterobasidium araucariae*	TULF-3	Tuskegee University	In preparation	Species
*Hydroporia lariciola*	TULF-1	Tuskegee University	In preparation	Genus+
*Kabatina thujae*	BS365-24-2	Colorado State University	In preparation	Genus+
*Peniophora crassitunicata*	TULF-13	Tuskegee University	In preparation	Species
*Neopestalotiopsis foedans*	GASo_Spr24_MR_1	Georgia Southern University	In preparation	Species
*Curvularia pseudobrachyspora*	GASo_Spr24_BC_2	Georgia Southern University	In preparation	Species
*Nigrospora vesicularifera*	GASo_Spr24_FS_1	Georgia Southern University	In preparation	Species
*Diaporthe infecunda*	GASo_Spr24_DR_1	Georgia Southern University	In preparation	Species
*Arcopilus aureus*	GASo_Spr24_DR_2	Georgia Southern University	In preparation	Species
*Pestalotiopsis microspore*	GASo_Spr24_JT_1C	Georgia Southern University	In preparation	Species
*Nigrospora macarangae*	GASo_Spr24_TB1B	Georgia Southern University	In preparation	Species
*Tillitiopsis* sp.	UGAMyco_010_2	University of Georgia	In preparation	Species
Tremellalales sp.	UGAMyco_012_3	University of Georgia	In preparation	Genus+
*Memnoniella longistipitata*	SM_2.1	Binghamton University	In preparation	Genus+
*Hypomyces robledoi*	HD_S.1A1	Binghamton University	In preparation	Genus+
*Vararia minispora*	RBS_01OO	Binghamton University	In preparation	Genus+
*Psathyrella carminei*	Gall_01_NG.10	Binghamton University	In preparation	Genus+
*Entosordaria quercina*	RBS_01NN	Binghamton University	In preparation	Genus+
*Ophiosphaerella korrae*	RSR_05.3	Binghamton University	In preparation	Genus+
*Neoantrodia serialis*	SM_1.6	Binghamton University	In preparation	Genus+
*Seiridium marginatum*	RBS_01A	Binghamton University	In preparation	Genus+
*Coprinellus christianopolitanus*	Gall_01_G.6	Binghamton University	In preparation	Genus+
*Xenocamarosporium acaciae*	HD_S.1B4	Binghamton University	In preparation	Genus+
*Ascochyta phacae*	RSR_02.9	Binghamton University	In preparation	Genus+
*Cubamyces menziesii*	SM_5.2	Binghamton University	In preparation	Genus+
*Phaeodiaporthe appendiculata*	Gall_01_NG.7	Binghamton University	In preparation	Genus+
*Paracamarosporium hawaiiense*	HD_S.4B3	Binghamton University	In preparation	Genus+

## Conclusion

The urgent need to increase knowledge in fungal biology and genomics requires targeted approaches focusing on workforce development and scientific discovery. Myco-Ed is effective at generating new fungal genomes while training a large number of students in fungal biology and genomics. These efforts directly contribute toward meeting the increasing need for expertise in bioinformatics and mycological research. The Myco-Ed program also has the potential to compile many novel, high-quality fungal reference genomes, especially for understudied clades, a goal that directly benefits fungal genomics and is highly relevant for a wide range of fields including ecology, evolutionary biology, medical biology, plant pathology, plant–fungal interactions, biochemistry, pharmaceuticals, and molecular biology.

## Supporting information

S1 Table Myco-Ed participating classrooms in 2023 and 2024 with respective institution, term, and number of students. (XLSX)
