# Correction: Myco-Ed: Mycological curriculum for education and discovery

**DOI:** 10.1371/journal.ppat.1013625

**Published:** 2025-10-24

**Authors:** Sara Branco, Peter G. Avis, Kerrie Barry, Scott Bates, Gerald M. Cobián, Ellen G. Dow, Sara Gremillion, Amy Honan, Chinyere A. Knight, Kurt LaButti, C. Alisha Quandt, Jane E. Stewart, Jayson Talag, Andrew W. Wilson, Lotus Lofgren, Stephen James Mondo

The authors, Sara Branco, Lotus Lofgren and Stephen James Mondo, should be noted as shared co-corresponding authors on this work. Stephen James Mondo’s email address is: sjmondo@lbl.gov.

## References

[1] Branco S, Avis PG, Barry K, Bates S, Cobián GM, Dow EG, et al. Myco-Ed: Mycological curriculum for education and discovery. PLoS Pathog. 2025;21(7):e1013303. doi: 10.1371/journal.ppat.1013303 40680048 PMC12273965

